# Assessing the range of deployment for an intra-hospital medical emergency team

**DOI:** 10.1007/s11739-025-04064-5

**Published:** 2025-07-24

**Authors:** Fabian Heinold, Onnen Moerer, Johannes Wieditz, Lars-Olav Harnisch, Thaddäus Struk

**Affiliations:** 1 Department of Anaesthesiology, University Medical Center Göttingen, Georg-August University of Göttingen, Robert-Koch-Str. 40, 37075 Göttingen, Germany; 2 Department of Medical Statistics, University Medical Center, Humboldtallee 32, 37073 Göttingen, Germany

**Keywords:** Medical, Emergency, Team (MET), Deployment protocol analysis, NACA, Score, Critical care response, Intrahospital emergency

## Abstract

Medical emergency teams (MET) play a crucial role in managing hospital emergencies. This single-center, retrospective cohort study at the University Medical Center Göttingen examines deployment patterns and operational challenges. Using the National Advisory Committee for Aeronautics (NACA) score, it assesses case severity, emergency care trends, and the influence of location and timing on patient outcomes. All MET calls handled by the Department of Anesthesiology from June 2019 to June 2023 were analyzed based on emergency documentation. 522 emergency protocols were analyzed. The most common reasons for MET activation were cardiovascular problems (32%), resuscitation (22%) and respiratory distress (15%). ROSC was achieved in 54.4% of CPR cases (62 out of 114 patients). On weekends, distribution of NACA scores shifted towards higher scores, OR = 1.831 (95% CI [1.298–2.583], *p* = 0.001). The severity of cases varied significantly between emergency locations (NACA score ≥ 5: public area: 1.3%, general ward: 56,8%, IMC: 65,2% and ICU: 86,8%, *p* < 0.001). There is a significant positive rank correlation of 0.566 (95%-CI [0.505–0.622], *p* < 0.001) between the NACA score and the duration of MET deployment. Severe emergencies (NACA 5–7) mainly occur on weekends and during late/night shifts, requiring specialized support. Higher NACA scores correlate with prolonged deployments, often exceeding 60 min. Optimizing alert systems and personnel planning is crucial for sustaining patient safety and care continuity.

**Supplementary Information:**

The online version contains supplementary material available at 10.1007/s11739-025-04064-5.

## Introduction

Medical emergency teams (MET) are essential in addressing emergencies and managing adverse events in hospitals. These teams can be called upon to treat critical patients, mitigating complications and improving outcomes. Their importance is emphasized by the demographic changes occurring across Europe, as university hospitals and maximum hospital care providers are expected to see an increase in the number of patients who are older and more severely ill in the coming years. This demographic shift is likely to result in higher hospital morbidity and mortality rates [1, 2]. Furthermore, the probability of adverse complications arising during medical procedures or inpatient treatment is anticipated to rise, primarily due to the increasing case complexity, which can be attributed to the accumulation of (subclinical) pathological conditions in older patients, along with their associated frailty [3]. Currently, the probability of adverse events occurring in hospitals ranges between 2.9 and 21.9%, with fatality rates associated with these events reaching up to 7.3% [4, 5]. Medical Emergency Teams have been proven to reduce mortality of patients experiencing adverse events [6, 7].

This study aims to evaluate the deployment patterns and operational efficiency of MET in managing in-hospital emergencies. By retrospectively analyzing the deployment protocols of 4 years the study investigates the severity of cases using the NACA score and explores the influence of timing and location on patient outcomes. The findings will provide insights into challenges faced by medical emergency teams operating in university hospital setting.

## Methods

In this single-center retrospective data analysis, in-hospital emergency interventions at the University Hospital Göttingen were evaluated for the period from July 1st, 2019, to June 30, 2023 after approval of the local ethics committee (application number 29/10/23; approved 17th October 2023).

At the University Hospital Göttingen, the Department of Anesthesiology holds the primary responsibility for delivering the Medical Emergency Team services and addressing on-site emergencies as well as aid with difficult airway management. Except of the Department of Internal Medicine, the affiliated ophthalmology clinic and parts of the Pediatric Clinic, the unit covers the entire public area, the general wards, as well as the functional suites, representing approximately 75% of the 1600-bed University hospital. The team consists of at least two members: a dedicated intensive care nurse and an anesthesiologist in his 3rd year of residency; the Medical Emergency Team can be summoned 24/7 via an emergency pager from anywhere in the hospital. Activation is possible from any hospital telephone and can be initiated by both medical and non-medical staff. In public areas, for instance, the MET may be activated via hospital security, the porter’s lodge, or by medical personnel who witness a medical emergency outside the hospital wards. During weekdays, the physicians on call for the MET are organized in a three-shift system, while the physicians operate in a two-shift system on weekends; nurses always operate in a three-shift model. The equipment and necessary devices are stored on a special emergency cart (Suppl. Picture 1) and all responses are documented on a dedicated protocol (Suppl. Picture 2).

The following data were extracted from the emergency documentation: Age, sex, location (Public area, General ward, Functional suites (including but not limited to CT, MRI, interventional radiology and endoscopy), Intermediate care unit and Intensive care unit) and time of the alert, the indication for the intervention, the outcome, pharmacological therapy and invasive procedures performed. The duration of the deployment was recorded in three categories: up to 30 min, 30–60 min, and > 60 min. Cases were correlated with the physicians ICU-shift they occurred in, using the documented time of alarm. For business days, cases were attributed to the early shift from 07:00AM to 03:59 PM, to the late shift from 04:00PM to 09:59 PM and to the night shift from 10:00 PM to 06:59 AM, respectively. On weekends and on holidays, cases were attributed to daytime-shift between 08:00 AM and 7:59 PM and attributed to the night shift from 08:00 PM to 07:59 AM if the following day was a weekend day or holiday, or to 06:59 AM if the following day was a business day. For cases involving cardiopulmonary resuscitation (CPR), it was additionally recorded whether the patient achieved return of spontaneous circulation (ROSC) during the intervention.

Subsequently National Advisory Committee for Aeronautics (NACA) Score was assessed from the available data by two experienced emergency providers [8, 9]. Briefly the NACA score is used to describe the severity of injuries, illnesses, or poisonings in emergency medicine. This system allows healthcare providers to assess and categorize the clinical condition, facilitating standardized communication and decision-making in emergency situations (Suppl. Table 1).

Data was collected in a spreadsheet (Microsoft Excel version 2021, Microsoft, WA, USA) and statistically analyzed using “R” (R Foundation for Statistical Computing, version 4.3.3,). Data were analyzed descriptively using mean (± SD) and numbers (percentages), as appropriate. Due to partially missing information, the total number of patients may vary in some graphics. The study did not account for false alarms, as they were not documented. To estimate the distribution of NACA levels, we fitted an ordinal model with working day [weekday; weekend] and shift-type [day shift; late/night shift] as covariates with corresponding variable interaction. Parameter estimates are provided with corresponding 95% confidence intervals. Tests are performed two-sided at a significance level of 5%.

## Results

### Number and location of deployment and NACA-score distribution

**Fig. 1:**
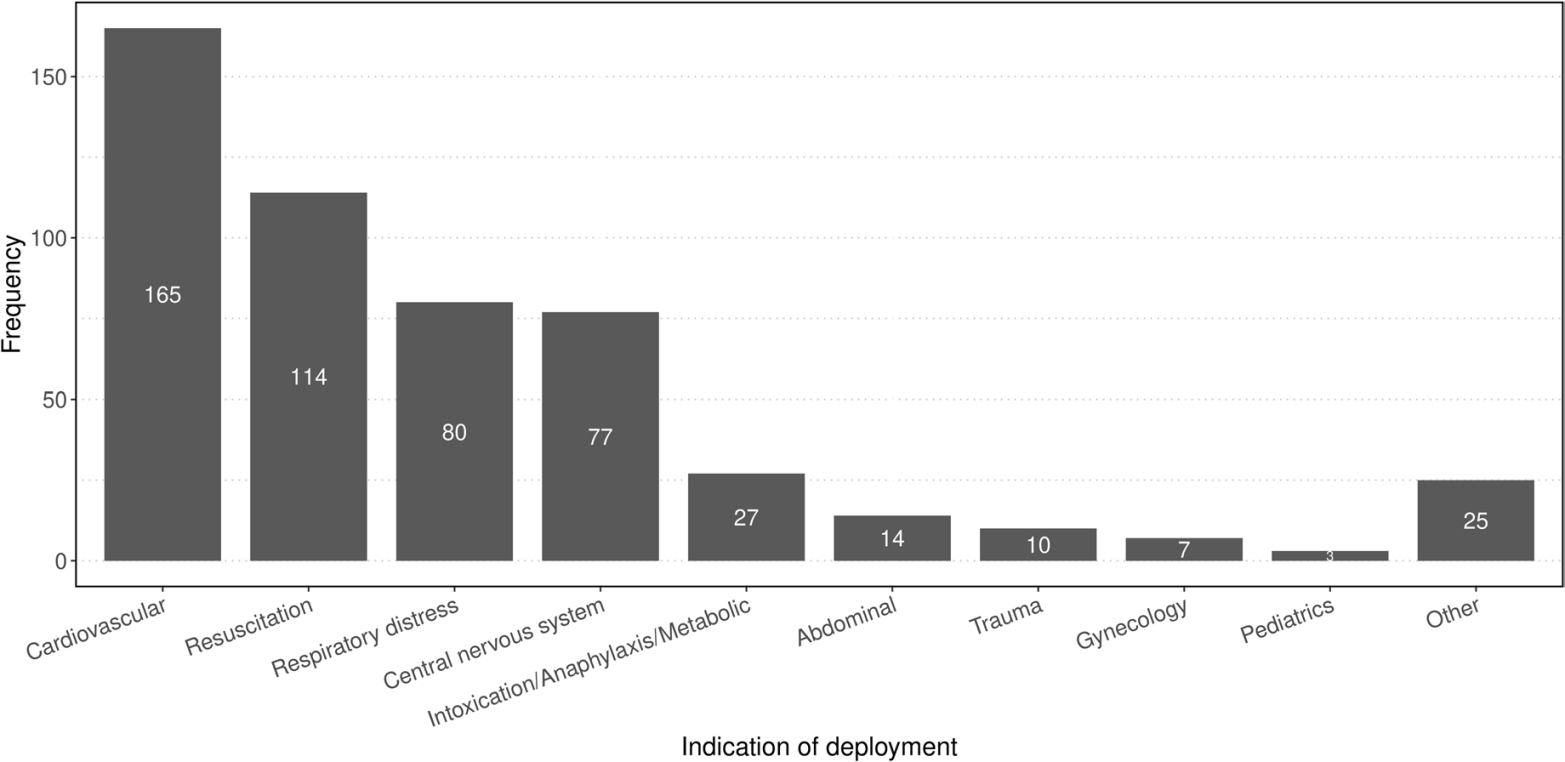
Indication of deployment

During the observation period, the MET was deployed 522 times, averaging approximately 0.36 deployments per day. A total of 53.2% male (*n* = 278) and 46% female patients (*n* = 240) and patients’ unknown sex (0,8%, *n* = 4) were treated; the median age was 66 years. The most common indication for deployment were cardiovascular problems at 32% (*n* = 165), followed by resuscitations at 22% (*n* = 114) and respiratory distress at 15% (*n* = 80). Pediatric cases were very rare, accounting for only 0.5% (*n* = 3), but were always severe with a NACA-Score above 4. Although the MET is not routinely deployed to the pediatric clinic during daytime hours, it may be called upon at night in cases of resuscitation or anticipated difficult airways, additionally, the MET may encounter emergencies of pediatric patients in the functional suites (Fig. 1).

Emergencies took place on general wards in 42.6% (*n* = 222) of cases, in 29.6% (*n* = 154) in the public area, 16.1% (*n* = 84) in functional suites, 4.4% (*n* = 23) in intermediate care units and 7.3% (*n* = 38) in intensive care units. In one case, the location was not documented. Life-threatening situations, defined by a NACA-Score of 5 or higher, within public places were exceedingly rare (1.3%). On general wards, a wide range of conditions was observed. In intensive care units the cases presented to the MET ranged from help with unexpected difficult airway to intra-hospital cardiac arrest. The serious pre-emergency status of the ICU patients contributed to the overall patient outcome of cases presented to the MET. On IMCs and in the functional suites, there was a more heterogeneous distribution of the NACA-Score, although the patients were more compromised compared to those in the public area but less than in the ICU as expected. Overall, an increase in the NACA-Score could be observed from the public area, through the general ward, up to the intensive care unit (Suppl. Figure 1).

**Fig. 2:**
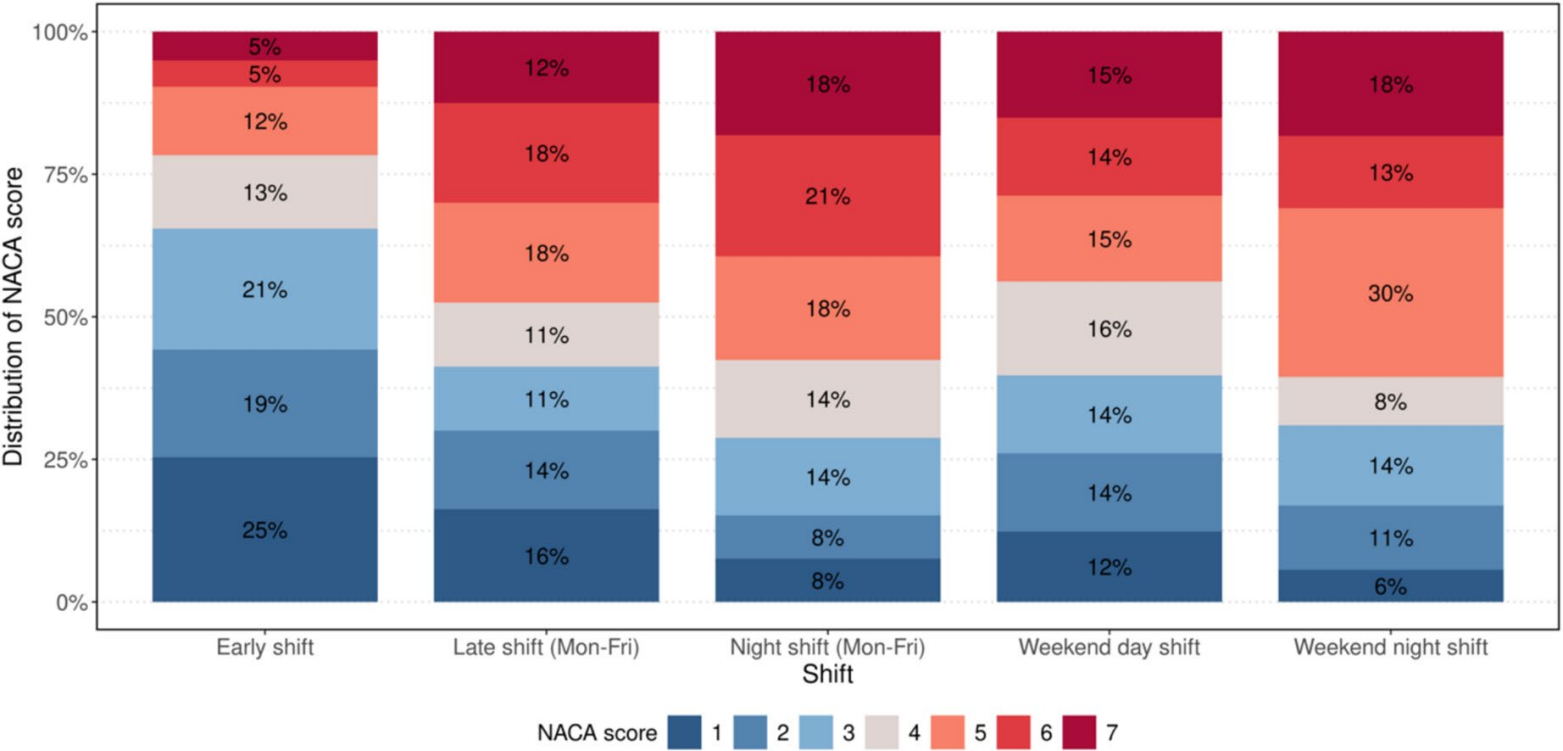
NACA-score distribution by shift

Examining the NACA-Score in relation to the time of alarm, a clustering of critical emergencies was observed at night and on weekends. During the early shift, 17% of patients had a NACA-Score of 5 to 6 points. This proportion increased to 36% during the late shift and remained high at 38% during the night shift on weekdays. A significant imbalance was also observed on weekends; during the weekend day shift, 29% of patients had a NACA-Score between 5 and 6 points, and this proportion rose to 43% during the late/night shift on weekends. Furthermore, it was observed that the proportion of patients who were declared deceased either upon arrival of the MET or after CPR efforts remained unsuccessful (NACA-Score 7) also increased depending on the time of day. During the early shift on weekdays, 5% of all treated patients were declared dead at arrival, while this number increased to 12% at night and 18% during the night shift on weekends (Fig. 2).

### Emergency medication, invasive procedures and CPR

**Fig. 3:**
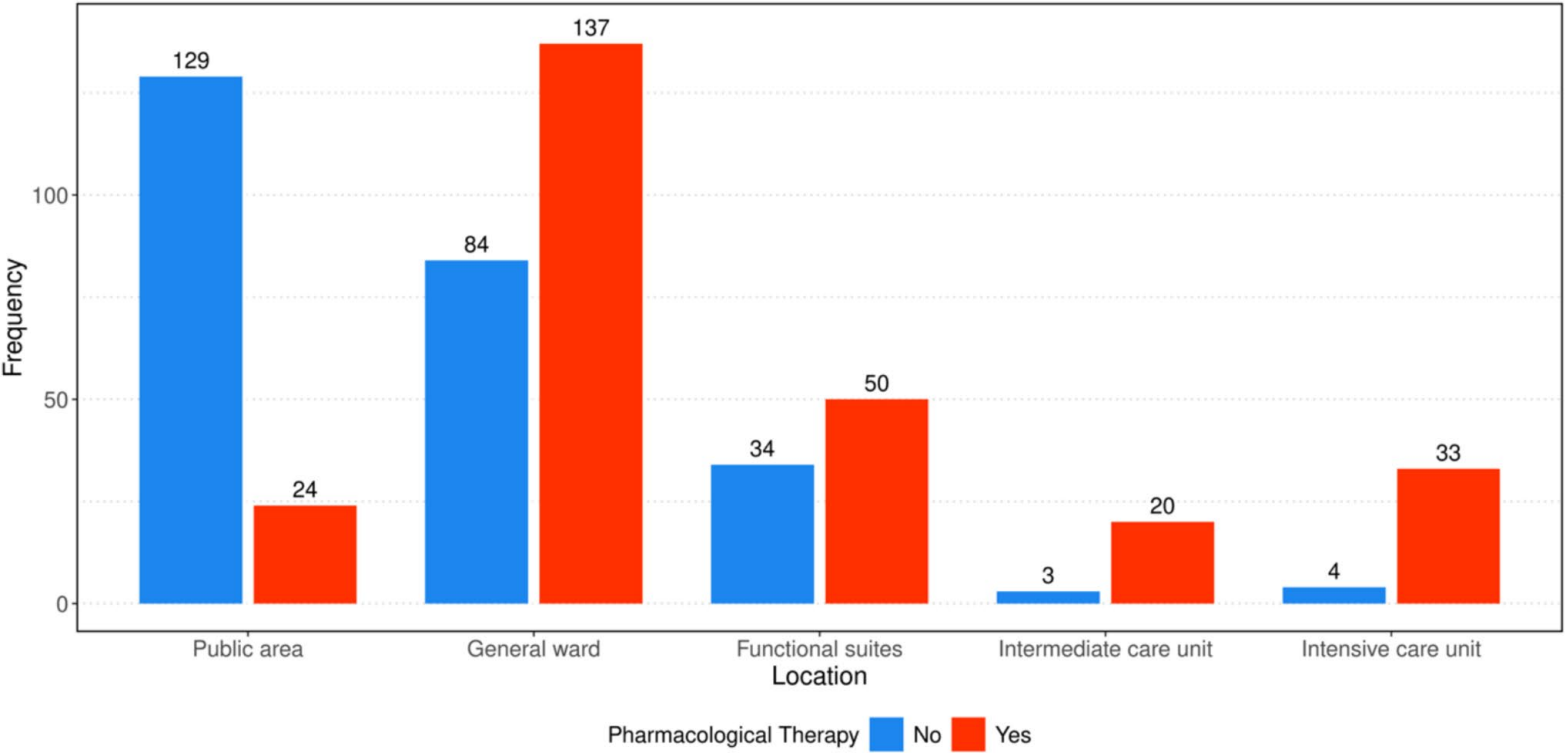
Pharmacological therapy by location

The need for interventions varied by site of incident. Emergency administration of medications was required in 85% of all emergencies during deployments to the ICU and in 89% of cases on the IMC. On the general ward, pharmacological therapy was needed in approximately 62% of cases, very similar to the functional suites where emergency medication was used in approximately 60% of cases. In contrast, it was indicated in only 16% in public areas. (Fig. 3). Overall, medication was authorized in nearly 50% (260/522) of all cases analyzed. Across all 522 interventions, intravenous vasopressors (adrenaline, noradrenaline, Akrinor®) were the most common pharmacological therapy used 151 times in 132 distinct emergencies. Endotracheal intubation was the most frequently performed invasive procedure in the examined cases, which had to be performed in a total of 136 cases (26% of all emergency responses). While 87% (*n* = 33) of the 38 patients in the ICU required intubation by the MET, it was necessary for 50% (*n* = 74) of patients on the general ward, and only 0.7% (*n* = 1) of the 154 patients in the public area (Suppl. Figure 2). Apart from oral intubations, other invasive measures were very rare; throughout the observation period, the MET performed a total of 3 cricothyrotomies and placed 5 chest drains. A difficult airway was documented in 27 intubations, accounting for 19.9% of all intubations. Consistent with the recorded NACA-Scores, the frequency of resuscitations also varied by location of emergency. In the public area, there was only one case of CPR, while the frequency of CPR was 14.3% (*n* = 12) in the functional suites, 44.7% (*n* = 17) in ICU, 33% (*n* = 8) in the intermediate care unit and 34.2% (*n* = 76) on the general ward. Among these, 62 out of 114 patients achieved ROSC, corresponding to a rate of 54.4%.

### Treatment results

In cases with a NACA score below 4, 87% (*n* = 134) of all patients in public areas underwent further evaluation in the emergency department, while 7% (*n* = 11) were discharged directly home. Admission to the operating room or the intensive care unit was rare in this setting, occurring in only 6% of cases (*n* = 9). Patients treated in the general ward were admitted to the ICU in 47% of cases (*n* = 104), with 19% (*n* = 42) dying before or during the deployment of the MET. Only 27% (*n* = 60) of the patients were able to remain on the ward and 7% (*n* = 42) were transferred to functional suites. Of patients treated in the ICU, 84,2% (*n* = 32) remained, 13,2% (*n* = 5) were declared dead, and only one patient, representing 2,6%, was immediately transferred to the OR.

### Time of deployment

**Fig. 4:**
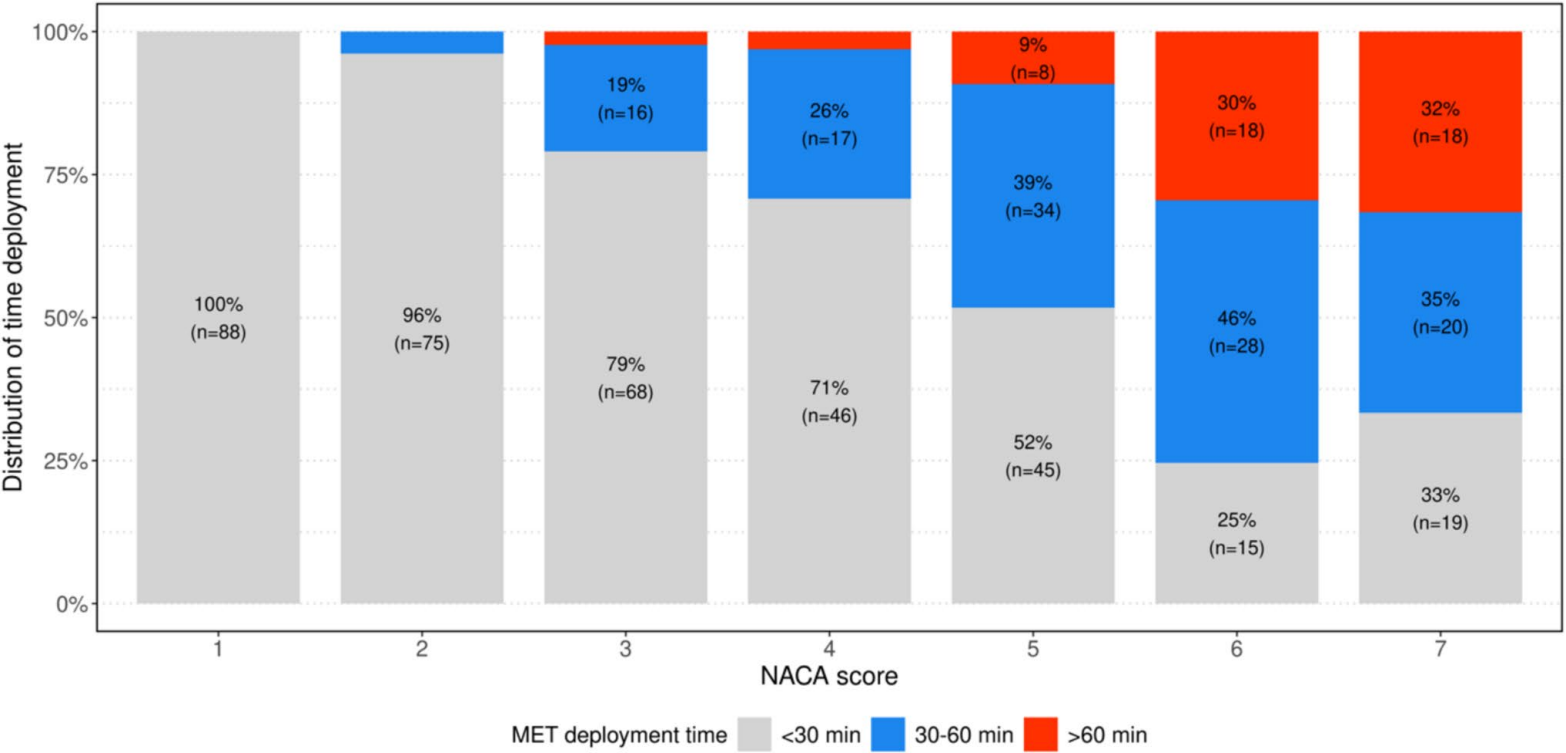
Time of deployment according to NACA-score

**Fig. 5:**
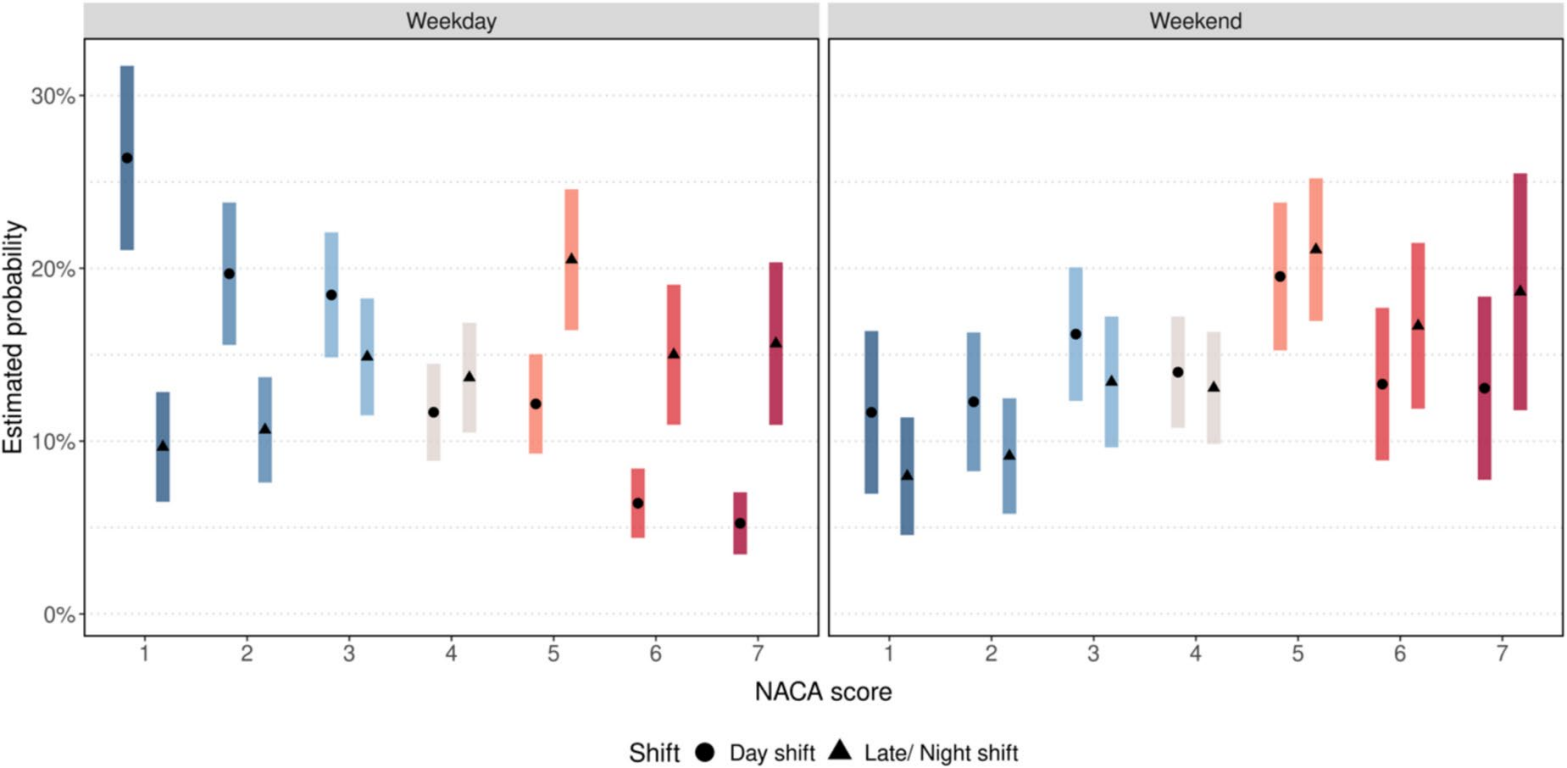
Estimated probability of NACA levels by shift and day type (Weekday vs. Weekend)

Overall, it is evident that the duration of deployments increases with a higher NACA-Score. While most cases with a NACA-Score of 1–2 were resolved within 30 min, the deployment time increases dramatically in more severe emergencies. In cases of resuscitation or patient death on the ward, the proportion of deployments lasting at least 30 min is considerably higher, accounting for 75% (*n* = 61) of all deployments with a NACA-Score of 6 and 67% (*n* = 57) with a NACA-Score of 7 (Fig. 4). There is a significant positive rank correlation of 0.566 (95%-CI [0.505–0.622], *p* < 0.001) between the NACA score and the duration of MET deployments, indicating that higher severity of patient conditions is associated with longer intervention times. Figure 5 demonstrates that more severe emergencies with higher NACA-Scores (5 to 7) predominantly occur during late and night shifts, regardless of whether they happen on weekdays or weekends. This trend is particularly pronounced for critical conditions, such as resuscitations (NACA 6) and deceased patients (NACA 7), where the probabilities are significantly higher during late shifts and night shifts, OR = 2.259 (95%-CI [1.597–3.190], *p* < 0.001). On weekends, the overall distribution of NACA-Scores shifts towards higher severity levels, with severe emergencies being more frequent compared to weekdays, OR = 1.831 (95%-CI [1.298–2.583], *p* = 0.001). Despite this imbalance, the difference between day and late/night shifts remains consistent, with severe cases being slightly higher during night hours, OR = 1.525 (95%-CI [0.858–2.710], *p* = 0.150). The model confirms statistically significant effects of shift timing (OR = 3.349, 95%-CI [2.287–4.924], *p* < 0.001) and weekends (OR = 2.713, 95%-CI [1.690–4.374]), as well as their interaction (OR = 0.455, 95%-CI [0.228–0.903], *p* = 0.025), providing critical insights for optimizing resource allocation and staffing during high-risk periods (Table 1; Fig. 5).

**Table 1:** Estimated probabilities [%] for NACA levels based on ordinal regression given day and shift of incident with corresponding 95% confidence intervals

NACA level	Weekday		Weekend	
	Day shift	Late/ Night shift	Day shift	Late/Night shift
1	26.4 [21.1–31.7]	9.7 [6.5–12.8]	11.7 [6.9–16.4]	8.0 [4.5–11.4]
2	19.7 [15.6–23.8]	10.7 [7.6–13.7]	12.3 [8.2–16.3]	9.1 [5.7–12.5]
3	18.5 [14.8–22.1]	14.9 [11.5–18.3]	16.2 [12.3–20.1]	13.4 [9.6–17.2]
4	11.7 [8.9–14.5]	13.7 [10.5–16.8]	14.0 [10.7–17.2]	13.1 [9.8–16.3]
5	12.2 [9.3–15.0]	20.5 [16.4–24.6]	19.5 [15.2–23.8]	21.1 [16.9–25.2]
6	6.4 [4.4–8.4]	15.0 [10.9–19.1]	13.3 [8.8–17.7]	16.7 [11.8–21.5]
7	5.2 [3.4–7.0]	15.6 [10.9–20.4]	13.1 [7.7–18.4]	18.6 [11.7–25.5]

## Discussion

Our results demonstrate that in-hospital emergencies are highly polarized: patients either present with non-life-threatening conditions, which was often the case when alarms were raised in public areas, or they were critically ill and required invasive interventions and pharmacological therapy. A significant proportion of activations, particularly in public areas, did not require direct intervention by the MET, as these cases were resolved without medication administration or invasive procedures. These patients were often transferred to the emergency department for further evaluation. The concentration of minor cases in public areas may stem from the liberal activation criteria in the in-hospital setting, allowing MET activation by any individual based on personal judgement. However, as indicated by Teuma and Trapani, early involvement of a rapid response team, even in seemingly less severe cases, can help promptly identify deteriorating patients and avert further complications [6]. Another key finding of this study is the significantly higher severity of emergencies during nights and weekends, as reflected by the clustering of high NACA-Scores (5–7) during these times. This aligns with previous studies, documenting poorer outcomes during off-peak hours, which may result from delayed recognition of emergencies and subsequent late MET activation [7, 10]. Consequently, the MET encounters a case-mix with disproportionately more severe cases during these high-risk periods. The implementation of additional on-call consultants, who holds a qualification in specialized intensive care medicine in anesthesiology, for high-severity cases (NACA-Score ≥ 4) during nights and weekends could be instrumental in addressing these challenges by providing additional expertise. Moreover, optimizing both alert systems and personnel planning in response to the distribution of serious emergencies could help maintain consistently high standards of patient care. Implementing a structured telephone triage could improve the assessment of disease severity, while the use of an Early Warning Score during evening shifts and on weekends may enable earlier detection of critically ill patients [11, 12].

This study also has limitations: First, data from the pediatric department as well as parts of the Department of Internal Medicine are missing, ultimately providing a limited view of all in-hospital emergencies that had occurred during the study period. While the NACA-Score was originally developed and validated for use in pre-hospital settings, it was retrospectively assigned in this study by two experienced physicians. Nevertheless, some degree of inter-individual variability in the assessment cannot be excluded. Although the NACA-Score has only been formally validated for pre-hospital use, its application in this context appears justifiable, as it reflects the severity of the emergency at the time of the MET team’s arrival, similar to the arrival of pre-hospital emergency care providers. This limitation should, however, be explicitly acknowledged when interpreting the findings. Our study may therefore serve as an initial derivation dataset for the use of the NACA-Sore in intra-hospital emergencies.

Additionally, the times documented in the protocols were only recorded approximately, limiting the granularity of time-based analyses. Moreover, the actual frequency of alerts could be underestimated, as there is no record of false alarms. The single-center design further restricts the generalizability of the findings. However, as the findings are in line with previously published work [7, 10], none of the limitations of this study restrict the validity of the results achieved.

This study also highlights the use of the MET for providing in-house assistance during critical interventions. This service is especially vital in ICUs not led by anesthesiologists, where physicians with advanced airway management skills may not always be present. By delivering specialized support, the MET also ensures that critically ill patients receive timely and effective care, bolstering the capabilities of other ICUs and maintaining patient safety during periods of limited staffing or expertise.

Additionally, we observed significant positive correlation between the NACA-Score and the duration of MET deployments, with higher-severity cases requiring notably longer intervention times. This is particularly evident during life-threatening emergencies, where extended MET involvement is often necessary for further diagnostics or patient stabilization. This is consistent with Dami and Darioli’s work, which linked higher NACA-Scores to more intensive interventions, elevated clinical risk and outcome [8].The duration of deployments was particularly prolonged during nights and weekends, further reflecting the complexity of cases during these times. Furthermore, this increases the burden on the responding teams, who face prolonged absences from their primary assignments. Such extended interventions reinforce the importance of strategic resource allocation and robust shift coverage to ensure patient safety and care continuity. Finally, to validate and expand these findings, conducting multi-center studies that include data from various medical centers, university hospitals, and METs led by various specialties, not only those managed by anesthesiologists, could provide broader insights into deployment patterns, the distribution of NACA-Scores, and their comparison with other established severity and early warning scores. Such research would help to further clarify the impact of case severity on patient outcomes and enhance the effectiveness of MET services across different healthcare settings.

## Supplementary Information

Supplementary material 1.

## Data Availability

The datasets generated and analyzed during this study are available from the corresponding author on reasonable request.
