# Supplementary material for: Assessing the range of deployment for an intra-hospital medical emergency team
Source: Intern Emerg Med. 2025 Jul 24;20(7):2103–10. doi: 10.1007/s11739-025-04064-5 (PMC12534359; doi:10.1007/s11739-025-04064-5)
Supplement: Supplementary file 1 — Supplementary material 1. [file 11739_2025_4064_MOESM1_ESM.docx]

**
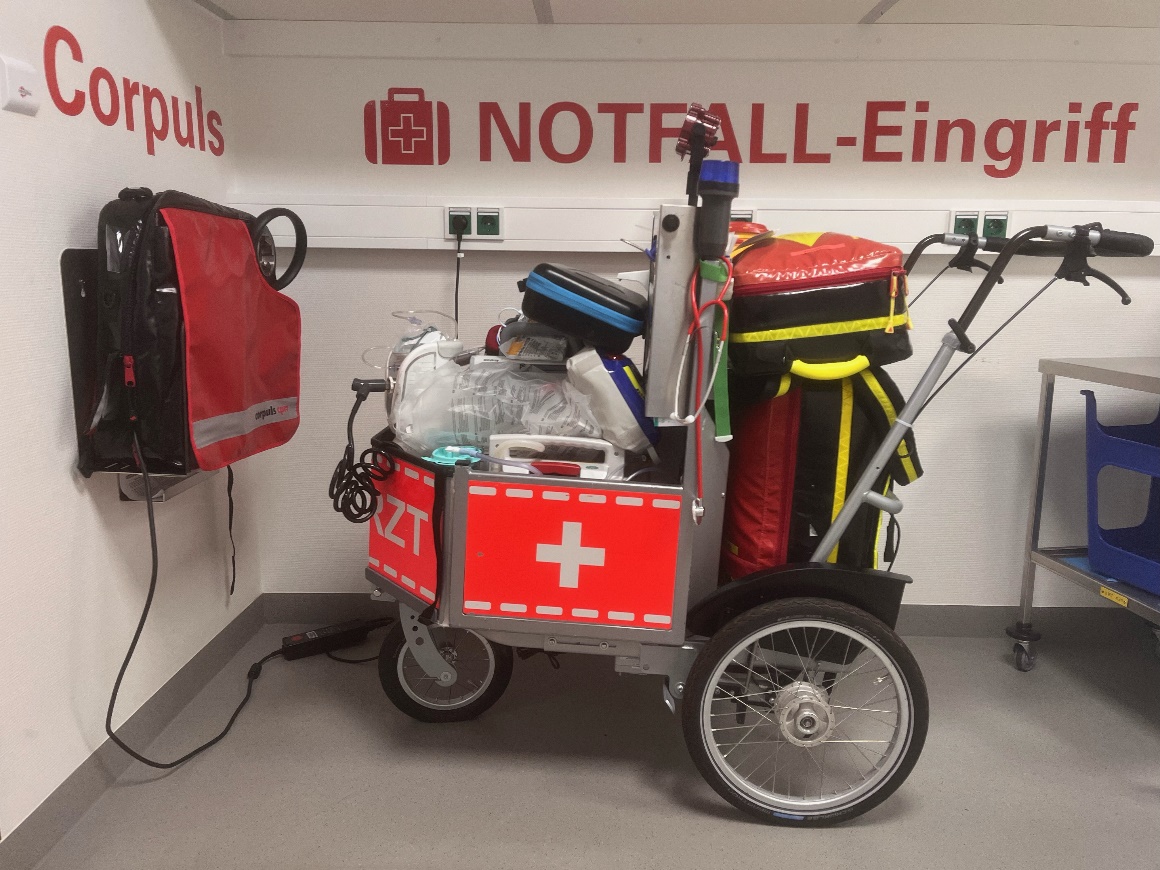
**

**Supl. Picture 1**: Emergency Cart with Equipment


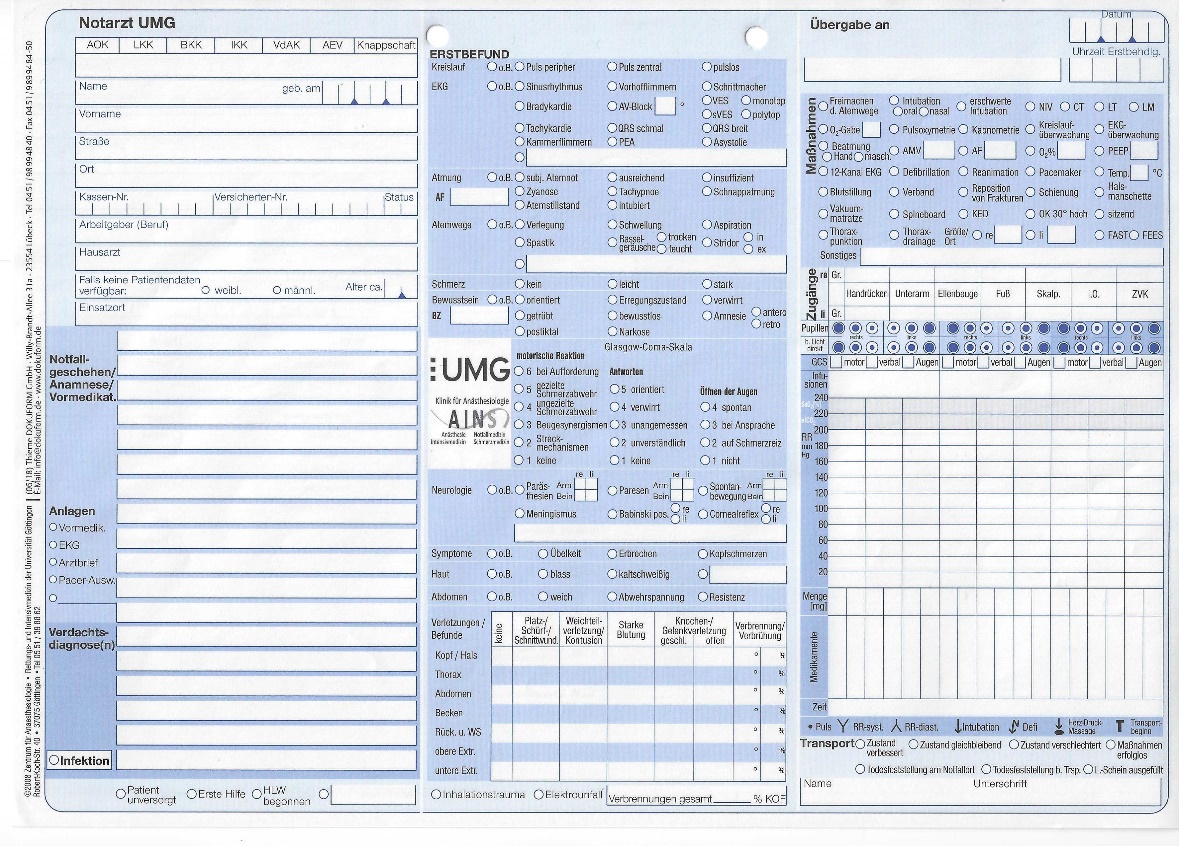


**Supl. Picture 2**: Emergency Protocol

**Supl. Table 1**: NACA-Score and Definition


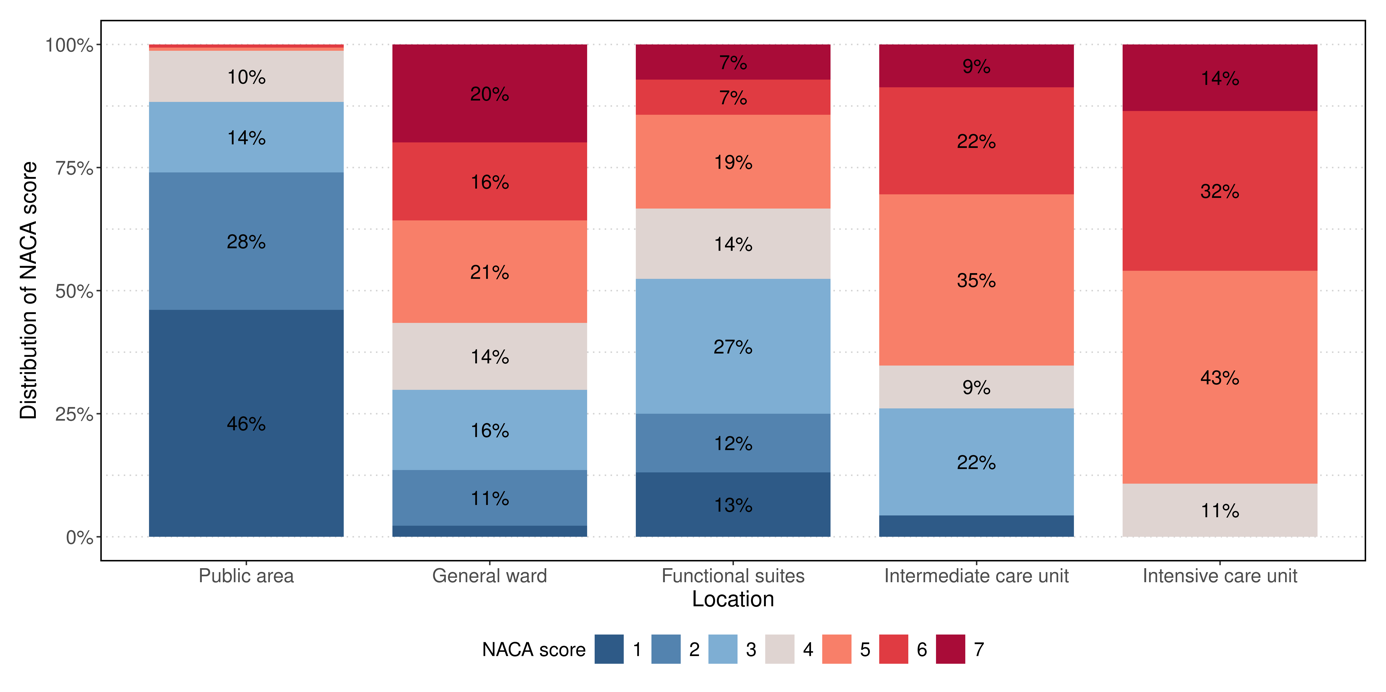


**Supl. Figure 1**: NACA-Score Distribution by Location


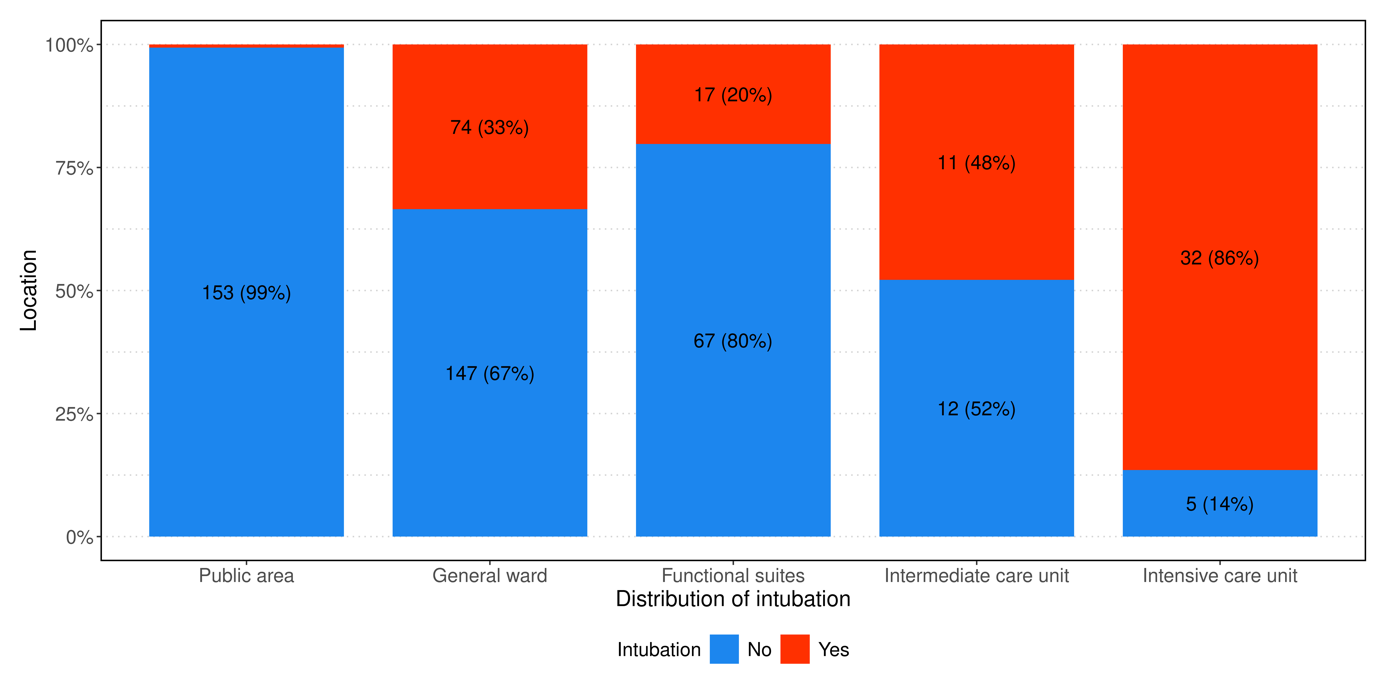


**Supl. Figure 2**: Intubation by location
